# Chronic Kidney Disease Increases Cerebral Microbleeds in Mouse and Man

**DOI:** 10.1007/s12975-019-00698-8

**Published:** 2019-05-04

**Authors:** Wei Ling Lau, Ane C. F. Nunes, Vitaly Vasilevko, David Floriolli, Long Lertpanit, Javad Savoj, Maria Bangash, Zhihui Yao, Krunal Shah, Sameen Naqvi, Annlia Paganini-Hill, Nosratola D. Vaziri, David H Cribbs, Mark Fisher

**Affiliations:** 1 Department of Medicine, Division of Nephrology, University of California, Irvine, CA USA; 2 Division of Nephrology and Hypertension, University of California-Irvine Medical Center, Suite 400, City Tower, 333 City Blvd. West, Orange, CA 92868 USA; 3 Institute for Memory Impairments and Neurological Disorders, University of California, 1111 GNRF, Irvine, 92697-4540 CA USA; 4 Department of Radiological Sciences, Neuroradiology, University of California, Irvine, CA USA; 5 Department of Cardiovascular Medicine, the Second Affiliated Hospital of Xi’an Jiaotong University, Xi’an, 710004 Shaanxi China; 6 Department of Neurology, University of California, Irvine, CA USA; 7 Departments of Anatomy & Neurobiology and Pathology & Laboratory Medicine, University of California, Irvine, CA USA

**Keywords:** Chronic kidney disease, Microbleeds, Mouse model, Endothelial cell culture, Brain MRI

## Abstract

Brain microbleeds are increased in chronic kidney disease (CKD) and their presence increases risk of cognitive decline and stroke. We examined the interaction between CKD and brain microhemorrhages (the neuropathological substrate of microbleeds) in mouse and cell culture models and studied progression of microbleed burden on serial brain imaging from humans. *Mouse studies:* Two CKD models were investigated: adenine-induced tubulointerstitial nephritis and surgical 5/6 nephrectomy. *Cell culture studies:* bEnd.3 mouse brain endothelial cells were grown to confluence, and monolayer integrity was measured after exposure to 5–15% human uremic serum or increasing concentrations of urea. *Human studies:* Progression of brain microbleeds was evaluated on serial MRI from control, pre-dialysis CKD, and dialysis patients. Microhemorrhages were increased 2–2.5-fold in mice with CKD independent of higher blood pressure in the 5/6 nephrectomy model. IgG staining was increased in CKD animals, consistent with increased blood–brain barrier permeability. Incubation of bEnd.3 cells with uremic serum or elevated urea produced a dose-dependent drop in trans-endothelial electrical resistance. Elevated urea induced actin cytoskeleton derangements and decreased claudin-5 expression. In human subjects, prevalence of microbleeds was 50% in both CKD cohorts compared with 10% in age-matched controls. More patients in the dialysis cohort had increased microbleeds on follow-up MRI after 1.5 years. CKD disrupts the blood–brain barrier and increases brain microhemorrhages in mice and microbleeds in humans. Elevated urea alters the actin cytoskeleton and tight junction proteins in cultured endothelial cells, suggesting that these mechanisms explain (at least in part) the microhemorrhages and microbleeds observed in the animal and human studies.

## Introduction

One of the most significant stroke neurology discoveries in recent years is the emergence of chronic kidney disease (CKD) as an independent risk factor for early cognitive decline and cerebrovascular disease (reviewed in [1]), especially cerebral microbleeds and stroke [2–9]. While CKD and cerebrovascular disease share common risk factors, CKD appears to have an impact that goes well beyond traditional risk factors such as hypertension and diabetes [10, 11]. Given the high prevalence (about 50%) of cerebral microbleeds and cognitive impairment in patients with advanced CKD [2–4, 6, 7], this relationship deserves further study.

Cerebral microbleeds are small foci of hemosiderin–iron demonstrable on magnetic resonance imaging (MRI), believed to reflect underlying cerebral microhemorrhages [12, 13], and indicative of heightened risk for stroke, both hemorrhagic and ischemic [9, 14]. Specific MRI sequences (gradient echo and susceptibility-weighted imaging) demonstrate these focal areas of signal loss in brain parenchyma measuring ≤ 10 mm [12, 15]. Cerebral microbleeds are age-dependent, with prevalence approaching 20% by age 65 [16]. In addition to age, hypertension and cerebral amyloid angiopathy are the best described risk factors for development of microbleeds [13, 17]. In late-stage CKD, microbleeds are present in up to 50% of the population [2–4].

Concurrently, cognitive impairment is both more prevalent and more severe at lower levels of kidney function [18], reaching a prevalence of 30–70% in chronic dialysis patients [6, 7]. In a cross-sectional analysis of 338 hemodialysis patients aged 55 years and older with age-matched controls, 34% of dialysis patients had severe cognitive impairment compared with 12% of controls [6]. Another 35% of the dialysis cohort had moderate cognitive impairment [6].

Several studies in non-CKD cohorts have demonstrated a strong association between cerebral microbleeds and declining cognitive function [19–22]. Epidemiologic data support co-existence of MRI microbleed burden and cognitive dysfunction in end-stage renal disease (ESRD) patients [8, 23]. Moreover, a recent report of 28 chronic dialysis patients with serial brain MRI showed an association between new microbleeds and decline in mini-mental state examination (MMSE) score [4]. Further, ESRD patients have a 3- to 4-fold higher incidence rate of both ischemic and hemorrhagic strokes compared with the general population [5]. In a cohort of Japanese hemodialysis patients who were stroke-free at baseline, the presence of cerebral microbleeds was an independent predictor of intracerebral hemorrhage during a 5-year follow-up period [9].

Here we report results from studies in mice and in CKD patients. We found increased brain microhemorrhages in CKD mice and describe impaired endothelial tight junction and actin cytoskeleton disruption as potential mechanisms for microhemorrhage formation in the CKD milieu. Retrospective analysis of serial brain MRI from non-CKD, pre-dialysis and chronic hemodialysis subjects confirmed ESRD as a significant risk factor for progression of microbleed burden.

## Methods

### Mice Experiments

#### Experimental Animals and Treatment Groups

**Fig. 1:**
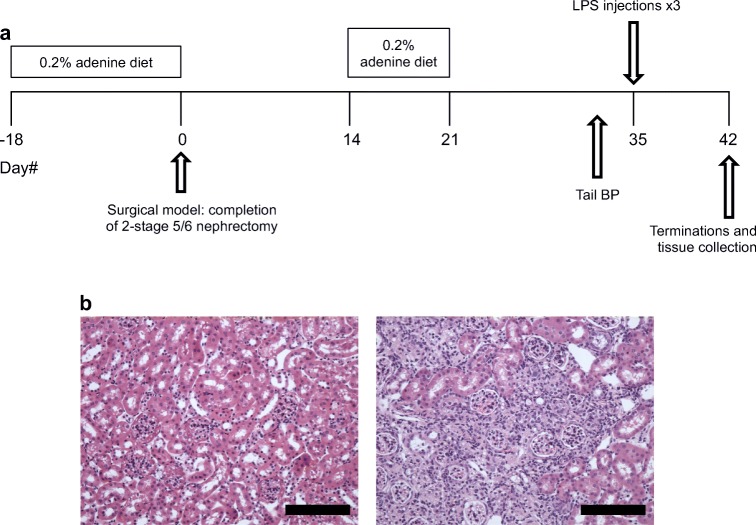
**a** Experimental timeline for chronic kidney disease (CKD) mice treated with lipopolysaccharide (LPS) injections to induce brain microbleeds. Adenine-CKD: Mice were placed on 0.2% adenine diet for 18 days, followed by regular chow for 2 weeks, then re-exposed to adenine diet for 1 week to maintain CKD. Two weeks after adenine re-exposure, LPS 1 mg/kg i.p. injections were given at 0, 6 and 24 h. Nephrectomy-CKD: 4.5 weeks after 5/6 nephrectomy, LPS 1 mg/kg i.p. injections were given at 0, 6, and 24 h. Subcutaneous saline hydration was given for 3 days after LPS injections. Tail blood pressure (BP) was measured prior to LPS injections. Mice were sacrificed 1 week after LPS injections. **b** Representative H&E stained kidney sections demonstrating adenine-induced tubulointerstitial injury CKD mice (right panel) compared with normal kidney from CTL animal (left panel), × 20 magnification. Scale bar = 100 μm

Two CKD mouse models were investigated. (1) Adenine tubulointerstitial nephritis model: Male C57BL/6J mice from Jackson Laboratories (Bar Harbor, ME) aged 10–12 weeks were fed a diet containing 0.2% adenine for 18 days to induce chronic interstitial nephropathy, placed back on regular chow for 2 weeks, and then re-exposed to adenine diet for 1 week to maintain CKD (Fig. 1a). Control mice were maintained on regular chow. (2) 5/6 nephrectomy model: Male C57BL/6J mice aged 10 weeks underwent two-stage surgery at Jackson Laboratories that involved left partial nephrectomy followed by right total nephrectomy 1 week later. Animals were delivered to the lab 1 week after the second surgery. These two models were utilized to determine effect of hypertension; hypertension is a hallmark of the 5/6 nephrectomy model, whereas adenine-CKD is non-hypertensive [24].

CKD animals were randomized to no lipopolysaccharide (LPS) or LPS injections. (In pilot studies, we determined that a longer duration of uremia was needed to detect higher burden of spontaneous microhemorrhages in non-treated CKD animals; mice on adenine diet for only 10 days beyond initial 18 days exposure had 3.0 ± 0.4 microhemorrhages per cm^2^.)

Five weeks after initial CKD induction and at an equivalent age in controls, mice were given intraperitoneal (i.p.) LPS injections (*Salmonella enterica* serotype typhimurium, L6511-10MG, Sigma-Aldrich, St. Louis, MO) to induce brain microbleeds. LPS was administered in three doses, 1 mg/kg at 0, 6, and 24 h [25]. LPS-treated mice were given hydration with subcutaneous saline injections two-to-three times per day for 3 days after LPS treatment. Blood pressure (BP) was measured 2 days before LPS injections via tail-cuff plethysmography (CODA-S2 multi-channel, Kent Scientific). All experiments were approved by the University of California, Irvine Institutional Animal Care and Use Committee.

#### Tissue Harvest and Blood Chemistries

Mice were euthanized 1 week after LPS injections or at an equivalent age in non-treated animals by exsanguination using cardiac puncture under inhaled isoflurane anesthesia. A 26-gauge needle was used as a cannula and inserted into the left ventricle, and ice-cold PBS solution was applied at a flow rate of 7–8 ml/min. After perfusion for 5 min, the left brain hemisphere was snap frozen for Western blot. The right brain hemisphere and both kidneys were fixed overnight in 4% paraformaldehyde and then stored in cold PBS prior to sectioning. Serum was aliquoted for blood chemistries. Blood urea nitrogen (BUN) was measured using the colorimetric kit from BioAssay Systems (Hayward, CA). Serum creatinine was measured using capillary electrophoresis at the O’Brien Kidney Research Core Center (UT Southwestern, Dallas, TX).

#### Detection of Microhemorrhages

The brains were mounted in 1.5% agarose and sectioned with a vibratome to generate coronal sections (40 μm). Every 5th section was collected for Prussian blue staining to detect microhemorrhages [26]. Prussian blue staining was performed using freshly prepared 5% potassium hexacyanoferratetrihydrate and 10% hydrochloric acid. Twenty minutes later, sections were rinsed in water and counterstained with nuclear fast red, dehydrated, and coverslipped. Microhemorrhages were identified at × 20 magnification as purple-blue deposits counted by three independent observers, and then the mean was calculated. Images of the observed positively stained sections were captured using a photomicroscope (Nikon Eclipse, Japan) for three animals per group to calculate microhemorrhage area. Whole slide images were scanned and the free ImageJ software (version 10.2) from the National Institutes of Health (www.imagej.nih.gov/ij/) was used to calculate total brain surface area. The number of microhemorrhages was normalized to total brain surface area per animal.

#### Western Blotting

Brain hemispheres for protein analysis were snap frozen at time of tissue collection and homogenized in ice-cold Tissue Extraction Reagent I (Thermo Fisher Scientific) supplemented with protease inhibitor cocktail (Roche Applied Science, Indianapolis, IN). Protein concentrations were measured with a BCA protein assay kit (Thermo Fisher Scientific). SDS-PAGE gel electrophoresis was done with 100 μg of protein per sample. Proteins were transferred onto PVDF membranes and then blocked for 1 h in 5% non-fat dry skim milk prepared in TBS-T buffer (10 mM Tris–HCl, 150 mM NaCl, and 0.1% Tween-20) and incubated with primary antibodies targeting claudin-5 (diluted 1:200, Sigma-Aldrich SAB4502981), occludin (diluted 1:200, Invitrogen 711500, Thermo Fisher Scientific), and normalized to GAPDH internal control (diluted 1:10,000, Abcam, Cambridge, MA) in 5% non-fat milk TBS-T overnight at 4 °C. The blots were then incubated with the respective anti-rabbit or anti-mouse secondary antibodies for 2 h at room temperature. After washing with TBS-T, bands were detected using the Luminescent Image Analyzer LAS-3000 (Fujifilm Life Science, Stamford, CT).

#### Immunohistochemistry

To detect blood–brain barrier (BBB) leakage, sections were incubated with biotinylated anti-mouse IgG secondary antibody at 1:100 for 1 h in room temperature (Vector Laboratories, Burlingame, CA, USA). Following washing with PBS, sections were incubated for 30 min with avidin–biotin–peroxidase complex (Vector Laboratories) at a dilution of 1:200. Staining was developed using 3′,3′-diamionbenzidine (DAB, Vector Laboratories) as chromogen. Percent area stained with IgG was analyzed using ImageJ software in non-LPS CTL and CKD brains. A threshold for positive IgG staining was chosen by manually evaluating a control animal for IgG staining, and this threshold was then kept equal for all included animals [27].

### Cell Culture Experiments

#### Brain Endothelial Cell Culture and Serum Treatment

Immortalized mouse bEnd.3 cells were purchased from the American Type Culture Collection (ATCC, Manassas, VA). Endothelial cell phenotype was confirmed via immunostaining for von Willebrand factor. Cell cultures were incubated in high-glucose complete Dulbecco’s modified Eagle’s medium (DMEM, ATCC 30-2002) containing 25 mM glucose supplemented with 10% fetal bovine serum (FBS) and 1% penicillin–streptomycin in a humidified incubator at 37 °C in an atmosphere of 5% CO_2_ and 95% air. Cells were used at passage 5 for all experiments. The cells were seeded at 5 × 10^6^ density on 12-well polyester Transwell inserts (0.4 μm pore, Costar) and confluence was achieved at 24–48 h. Culture medium was then changed to expose cells to different concentrations of FBS, human normal serum (HNS), or uremic serum (CKD) from dialysis patients. TEER readings were measured using the EVOM2 volt/Ohm Meter (World Precision Instruments, Sarasota, FL) at three locations per well to obtain an average. Experiments continued for 21 days, and culture medium was refreshed every 3–4 days. NHS and uremic serum were from previously banked samples obtained after IRB approval and informed consent.

#### Urea Cell Culture Experiments

To examine effects of urea (the most abundant retained toxin in CKD), bEnd.3 cells were incubated in high glucose DMEM with 10% FBS alone or in medium supplemented with 42 or 72 mg/dL (70 or 120 μm) urea (Sigma-Aldrich). These urea concentrations approximate the pre- and post-hemodialysis values generally found in ESRD patients, and are considered to be clinically relevant [28]. At the conclusion of a 24-h incubation period, the TEER was measured and cells were harvested and processed for Western blot analysis. For visualization of the actin cytoskeleton, bEnd.3 cells were grown on glass cover slips and exposed to the above urea concentrations for 24 h, fixed for 10 min in chilled 4% formalin/PBS and then processed for immunofluorescence staining using acti-stain 488 fluorescent phalloidin with DAPI nuclear stain (catalog# PHDG1-A, Cytoskeleton Inc., Denver, CO). Triplicate slides were done per group, and three frames were imaged per slide on ImageJ software to calculate area of phalloidin staining normalized to DAPI area.

#### Western Blotting

bEnd.3 cells from the urea experiments were pelleted, then lysed in Tissue Extraction Reagent I (Thermo Fisher Scientific) supplemented with protease inhibitor cocktail (Roche Applied Science). Protein concentrations were measured with a BCA protein assay kit, and Western blotting was done as described above with claudin-5 (diluted 1:500) and occludin (diluted 1:500) normalized to GAPDH internal control (diluted 1:20,000). Western blots were repeated at least three times for each sample.

### Human Studies

#### Retrospective Brain MRI Chart Review

Electronic medical records between January 1, 2008 and December 31, 2014 at University of California-Irvine Medical Center were screened using the Honest Broker system and UCReX (University of California Research Exchange) after IRB approval. Pre-dialysis CKD (*n* = 8) and chronic hemodialysis patients (*n* = 9) who had at least two brain MRI scans at separate time points were identified (of 10 hemodialysis patients initially identified, 1 was removed from final analysis due to missing images in the radiology PACS system). Controls with normal kidney function (*n* = 10) were manually matched to the hemodialysis CKD patients by gender and age ± 5 years.

#### Review of MRI for Cerebral Microbleeds

Microbleeds were counted by an attending neuroradiologist (DF) and analyzed for progression over time. Brain MRI with T2*-weighted and susceptibility-weighted imaging (SWI) and FLAIR (fluid attenuated inversion recovery) images were performed on 1.5T and 3T MRI scanners. Layer thickness for SWI sequences was performed at 2 mm, with an interlayer interval of 0. Microbleeds were counted based on 2-mm axial SWI. FLAIR was performed to detect white matter lesions at 3 mm slice thickness, also with an interlayer interval of 0.

#### Statistical Analysis

There were no data outliers upon screening with the Grubbs’ test (extreme studentized deviate method, http://graphpad.com/quickcalcs/grubbs1/). Differences among groups in mouse and in man were compared by chi-square (Fisher’s exact) tests for categorical variables and *t* tests and ANOVA for continuous variables. Continuous data are reported as mean ± SEM. For mouse data, we performed both a Kruskal–Wallis non-parametric and a one-way ANOVA with Tukey HSD tests. Two-way ANOVA (CKD-yes/no and LPS-yes/no) was used to test CKD interaction. Differences among groups were considered significant if *P* < 0.05. Figures were generated using GraphPad Prism 4 software (GraphPad Software, San Diego CA).

## Results

### Survival with LPS Treatment

CKD mice treated with LPS had a 80% suvival rate. Only mice that survived to 1 week after LPS treatment were included in the final analyses. All mice in other groups survived to the end of the experiment.

### CKD Significantly Increased Brain Microhemorrhages Burden After LPS Treatment

Animals with adenine-induced and 5/6 nephrectomy CKD showed significantly elevated blood urea nitrogen (BUN) and serum creatinine values compared with CTL animals (Table 1). Tail BP was significantly higher in nephrectomy-CKD animals compared with CTL and adenine-CKD animals (Table 1); however, microbleed counts in the two CKD groups were similar. H&E staining confirmed adenine-induced tubulointerstitial injury in the kidneys from CKD mice (Fig. 1b).

**Table 1:** Animal studies

Group	n	Body weight at termination (g)	Tail systolic BP (mmHg)	Serum creatinine (mg/dL)	Microhemorrhage quantitation (count per cm^2^)
CTL	8	29.7 ± 0.5	111 ± 3.7	0.1 ± 0.001	2.0 ± 0.5
				BUN: 33.2 ± 1.7	
CTL+LPS	11	28.9 ± 0.5	--	0.09 ± 0.002	3.6 ± 0.4
Ad-CKD	9	26.6 ± 0.3 ^#^	113 ± 4.3	0.42 ± 0.019 ^#^	4.5 ± 0.9
				BUN: 99.5 ± 8.2 ^#^	
Ad-CKD+LPS	11	25.1 ± 0.6 ^#^	--	0.29 ± 0.008 ^#^	10.3 ± 1.5 ^#^*
Neph-CKD	5	25.2 ± 0.5 ^#^	127 ± 3.3 ^#^	0.27 ± 0.022 ^#^	4.2 ± 1.1
				BUN: 124 ± 15 ^#^	
Neph-CKD+LPS	10	26.8 ± 0.6 ^#^	--	0.28 ± 0.022 ^#^	8.8 ± 2.5 ^#^
ANOVA p-value		< 0.05	< 0.01	< 0.05	< 0.01

Body weights, tail blood pressure (BP), serum biochemistries including BUN (blood urea nitrogen, mg/dL) and microhemorrhage quantitation of control (CTL) and chronic kidney disease (CKD) mice treated with intraperitoneal lipopolysaccharide (LPS) to induce brain microhemorrhages. Two models of CKD were examined: adenine tubulointerstitial nephritis (Ad-CKD) and 5/6 nephrectomy (Neph-CKD). Data shown as mean±SEM, ^#^*P*<0.05 compared with CTL, *P<0.05 compared with CTL+LPS

**Fig. 2:**
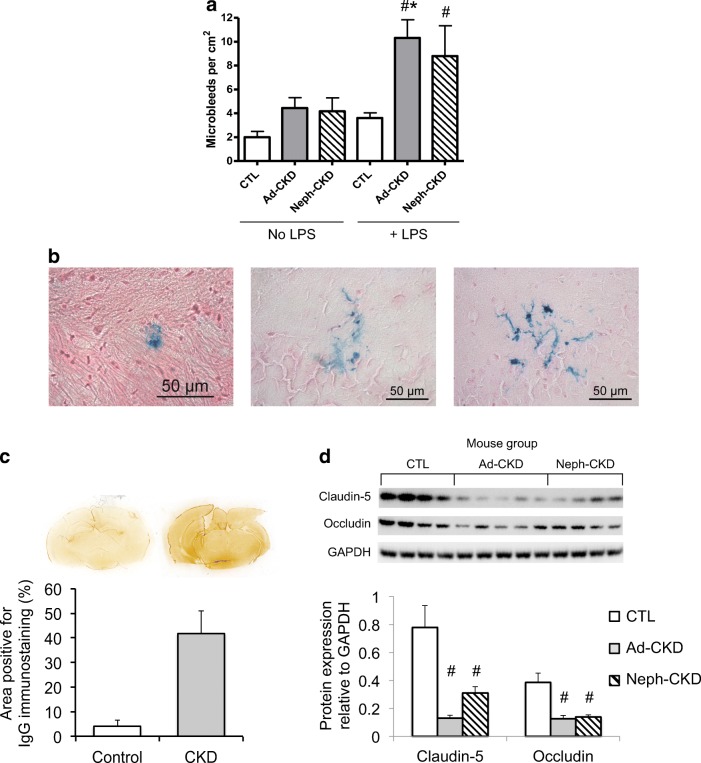
**a** Brain microhemorrhage burden was doubled in CKD mice compared with CTL animals, and LPS treatment increased microhemorrhage count. ^#^*P* < 0.05 compared with CTL, *P < 0.05 compared with CTL + LPS (data analyzed using one-way ANOVA). **b** Representative brain micrographs demonstrating parenchymal microhemorrhage in a CTL mouse (left), CTL + LPS mouse (middle), and adenine-CKD + LPS mouse (right), × 40 magnification. **c** Representative coronal brain sections from control (left) and CKD (right) mice. Increased blood–brain barrier permeability in CKD animal was evidenced by increased IgG staining. **d** Western blot data demonstrating decreased expression of tight junction proteins in brain tissue lysates from two CKD mouse models, adenine tubulointerstitial nephritis (Ad-CKD) and 5/6 nephrectomy (Neph-CKD) (*P* < 0.05 compared with controls, CTL)

Mean brain microhemorrhage burden was 2.0 ± 0.5 per cm^2^ in non-LPS CTL mice and was increased 2–2.5-fold in non-LPS CKD animals (adenine-CKD mice 4.5 ± 0.9 per cm^2^; nephrectomy-CKD mice 4.2 ± 1.1 per cm^2^) (Table 1; Fig. 2a). For the non-LPS groups, increase in microhemorrhages was significant for adenine-CKD compared with CTL but not for nephrectomy-CKD. LPS treatment increased microhemorrhage formation to the same degree in CTL and CKD animals by approximately 2-fold, *P* = 0.004. Microhemorrhages were predominantly distributed in the cerebellum, cortex, and sub-cortex as described previously [26]. The adenine-CKD + LPS group had the highest mean microhemorrhage count of 10.3 ± 1.5 per cm^2^. Morphology and size distribution of microhemorrhages were similar between CTL and CKD animals (Fig. 2b) with a trend for larger average microhemorrhage area in LPS-treated CKD mice (0.001 mm^2^ in CTL, 0.005 mm^2^ in CTL + LPS, 0.004 mm^2^ in adenine-CKD, 0.013 mm^2^ in adenine-CKD + LPS, 0.004 mm^2^ in nephrectomy-CKD, and 0.021 mm^2^ in nephrectomy-CKD + LPS, *P* = 0.24).

BBB function was evaluated by mouse IgG staining in the non-LPS animal groups. The percent area stained with IgG above the pre-set threshold intensity was significantly increased in CKD animals, 42 ± 9% compared with 4 ± 3% in CTL mice (Fig. 2c). Expression of claudin-5 and occludin tight junction proteins were significantly decreased in the brains of CKD mice (Fig. 2d).

### Exposure to Uremic Serum Decreased TEER and Tight Junction Protein Expression

**Fig. 3:**
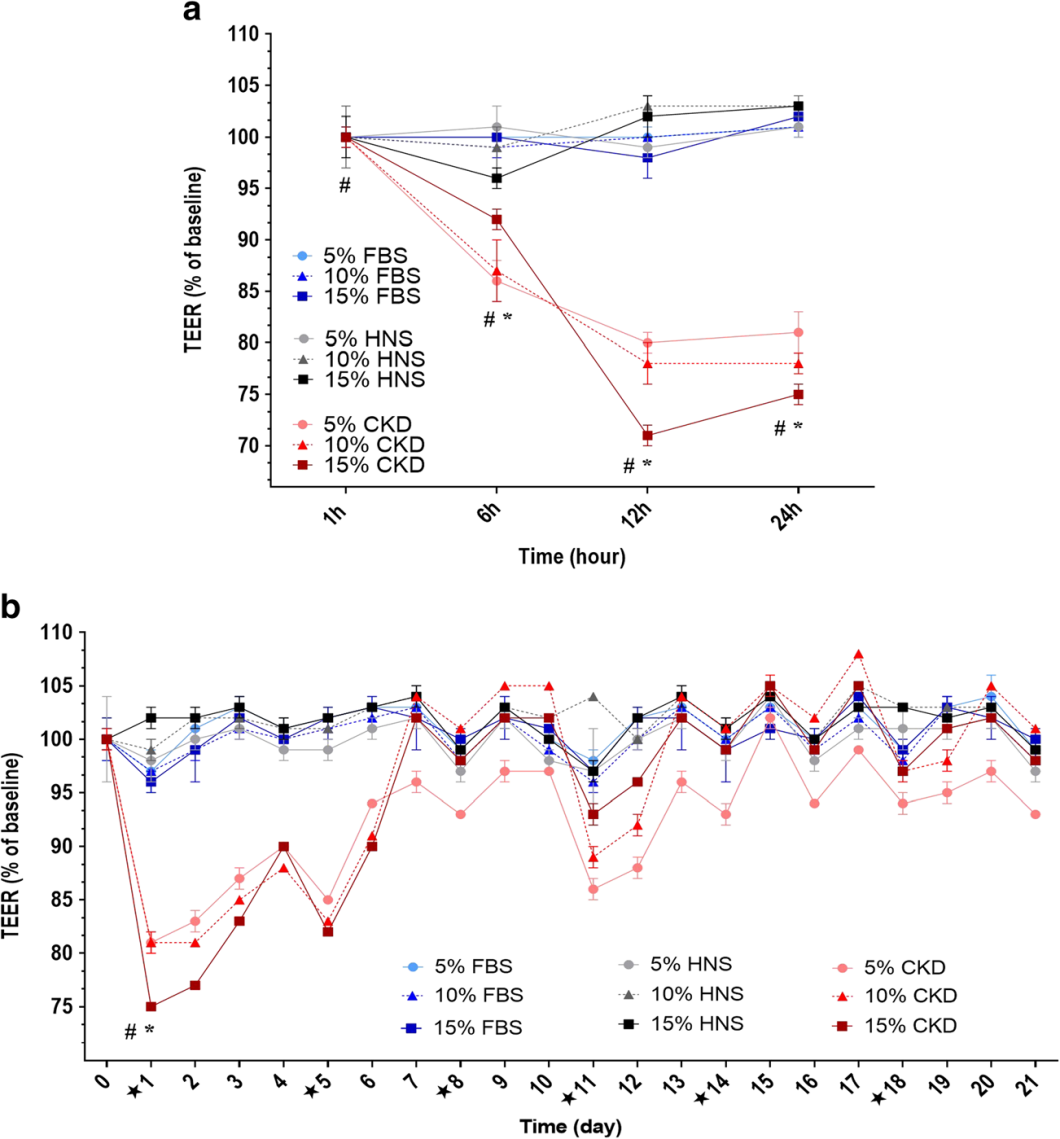
Incubation of bEnd.3 mouse brain endothelial cells with 5%, 10%, or 15% of fetal bovine serum (FBS) vs. human normal serum (HNS) vs. chronic kidney disease (CKD) serum from hemodialysis patients. **a** Treatment of bEnd.3 cells with CKD serum produced a marked drop in trans-endothelial electrical resistance (TEER) that was most pronounced at 12 h. Data are mean ± SEM; ^#^*P* < 0.05 in CKD compared with FBS groups, *P < 0.05 in CKD compared with HNS groups, ^**†**^*P* < 0.05 across CKD groups (dose effect). **b** The experiment was extended to 21 days to study chronic effects of serum treatment, and culture medium was refreshed every 3–4 days. The asterisk notation next to day number on the *x*-axis indicates days on which medium was refreshed. TEER values in the CKD groups showed an increase over time, suggesting repair of tight junction barrier under chronic uremic conditions. However, TEER remained significantly lower at 21 days compared with the FBS groups

Treatment of bEnd.3 cells with 5%, 10%, or 15% CKD serum from hemodialysis patients produced a marked drop in trans-endothelial electrical resistance (TEER) that was most pronounced at 12 h, when TEER was 74%, 69%, and 61% in the 5%, 10%, and 15% CKD groups respectively compared with 10% FBS control values (Fig. 3a), i.e., there was a dose-dependent TEER decline.

The chronic effects of serum treatment (21 days) (Fig. 3b) showed an increase in TEER values in the CKD groups over time suggesting repair of the junctional barrier. However, the TEER readings in all three CKD groups remained significantly lower at 21 days compared with the FBS groups (for example, 21-day TEER for the 5% CKD group was 88% of the TEER noted in 5% FBS group).

### Exposure to Elevated Urea Alone Decreased TEER in Conjunction with Actin Cytoskeleton Derangements and Decreased Tight Junction Protein Expression

**Fig. 4:**
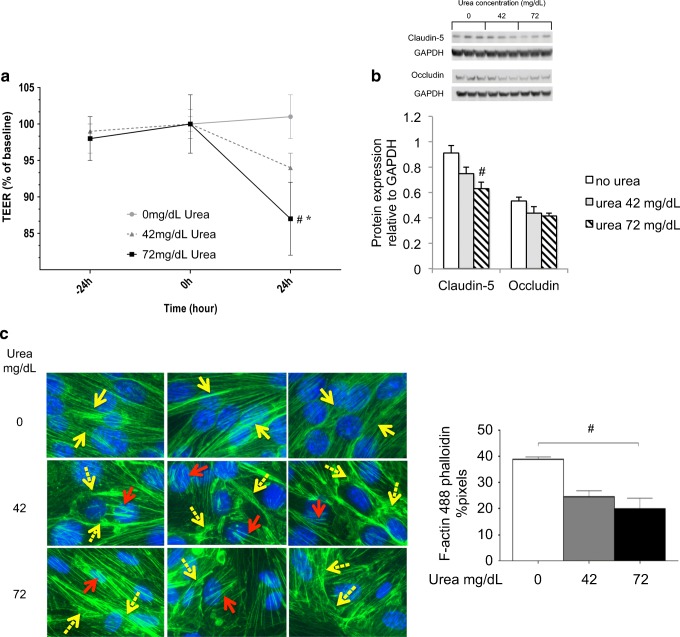
Treatment of bEnd.3 mouse brain endothelial cells with 0, 42, or 72 mg/dL of urea for 24 h. **a** Trans-endothelial electrical resistance (TEER) was significantly decreased with the 72 mg/dL urea exposure. ^#^*P* < 0.05 (0 h vs. 24 h); *P < 0.05 (− 24 h vs. 24 h). **b** There was dose-dependent decrease in expression of the tight junction proteins claudin-5 and occludin with increasing urea concentrations, one-way ANOVA *P* < 0.05 for the claudin-5 dataset and *P* = 0.1 for the occludin dataset. ^#^*P* < 0.05 compared with 0 mg/dL urea group. **c** bEnd.3 cells exposed to urea demonstrated derangement and decreased expression of the F-actin cytoskeleton. Immunofluorescence staining done with acti-stain 488 fluorescent phalloidin (green pseudo-color) and DAPI nuclear stain (blue). Yellow arrows represent cortical fibers, dashed yellow arrows represent fragmented cortical fibers, and red arrows represent radial stress fibers. ^#^*P* < 0.05 compared with 0 mg/dL urea group

Treatment of confluent bEnd.3 cells with 0, 42, or 72 mg/dL urea in DMEM with 10% FBS resulted in a concentration-dependent drop in TEER (Fig. 4a). The tight junction proteins claudin-5 and occludin were decreased ~ 25% after 24-h exposure to the highest urea concentration, with the drop in claudin-5 reaching statistical significance (Fig. 4b). Urea exposure was associated with disruption of the actin cytoskeleton as visualized by phalloidin immunofluorescence (Fig. 4c). Phalloidin staining was significantly decreased in the 72 mg/dL urea group compared with the reference zero urea group (*P* < 0.05). Cells remained viable as evidenced by unchanged trypan blue exclusion and maintained endothelial phenotype on immunostaining for von Willebrand factor.

### Microbleed Progression on Brain MRIs Was Significant in Chronic Hemodialysis Patients

**Fig. 5:**
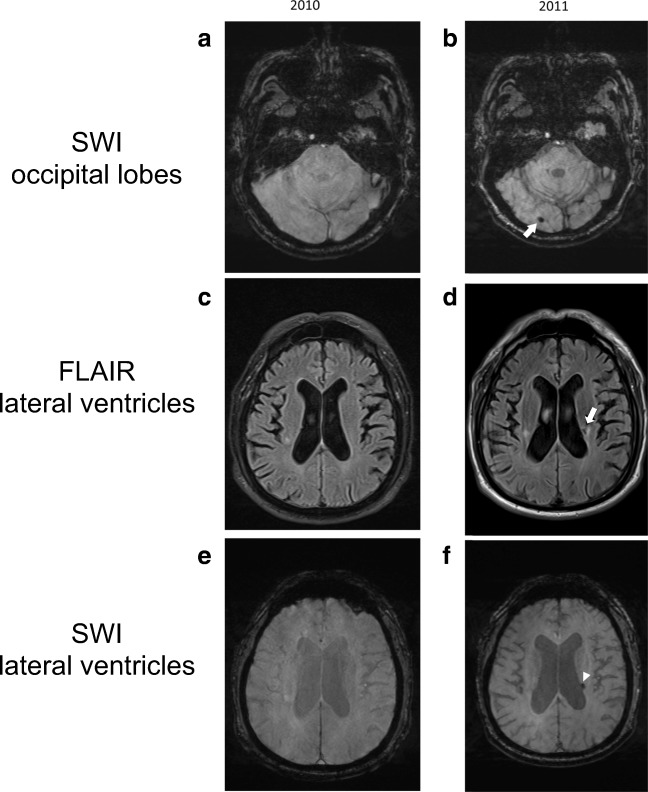
Representative magnetic resonance images showing progression of brain pathology in a chronic hemodialysis patient. The patient had imaging done in 2010 (images A, C, and E) and in 2011 (images B, D, and F). (A, B) SWI (susceptibility-weighted imaging) at the level of the occipital lobes demonstrate an interval microbleed within the right occipital lobe (white arrow). (C, D) FLAIR (fluid attenuated inversion recovery) images at the level of the lateral ventricles demonstrate an interval lacunar infarction of the left periventricular white matter (white arrow). (E, F) SWI images through the lateral ventricles demonstrate blood products associated with this lesion, compatible with a hemorrhagic infarction or microinfarction

In a retrospective analysis of serial brain MRIs, one third to half of the CKD cohorts (4/8 of pre-dialysis and 3/9 of dialysis participants) had microbleeds on the first MRI compared with 1/10 patients in the non-CKD group (Table 2). From chart review, most of the ESRD cases were due to diabetic nephropathy and hypertension (4); other causes included lupus nephritis (2), hypertension (1), IgA nephropathy vs. lupus nephritis (1), and cystinosis (1). Figure 5 shows representative brain MRI images (SWI and FLAIR) from a chronic hemodialysis patient who developed new microbleed and lacunar infarct lesions on follow-up imaging.

**Table 2:** Human studies retrospective analysis of serial MRIs demonstrated progression of microbleed burden in 3/9 chronic hemodialysis patients vs. 0/10 and 0/8 in the non-CKD controls and pre-dialysis CKD cohorts respectively (^†^*P* = 0.03 per Fisher’s exact test). Data shown as mean ± SEM where appropriate

Group	*n*	Age (years) % Male	Hypertension comorbidity	No. of microbleeds on first and second MRI	No. of months follow-up	No. of patients with microbleed progression
Controls	10	55.5 ± 5.3 20%	30%	1 ➔ 1 (*n* = 1) 0 ➔ 0 (*n* = 9)	19.5 ± 5.6	0 of 1
Pre-dialysis CKD	8	63.1 ± 4.4 50%	75%	1 ➔ 1 (*n* = 3) 2 ➔ 2 (*n* = 1) 0 ➔ 0 (*n* = 4)	16.0 ± 3.3	0 of 4
Dialysis CKD	9	52.6 ± 6.4 11%	89%	7 ➔ 7 (*n* = 1) 7 ➔ 13 (*n* = 1) 12 ➔ 13 (*n* = 1) 0 ➔ 268 (*n* = 1) 0 ➔ 0 (*n* = 5)	18.4 ± 3.9	3 of 4^†^

^†^*P* = 0.03 Fisher’s exact test for proportion with microbleed progression

Two of three dialysis patients showed progression of microbleed burden at follow-up. One dialysis patient who had zero microbleeds on first MRI had new microbleeds on follow-up imaging. In contrast, microbleed number was unchanged in all pre-dialysis patients. The difference in proportion that progressed over follow-up period among the three groups was statistically significant (*P* = 0.03). There was no statistically significant difference in mean age, mean follow-up months, or proportion that were male, but power was limited by the small sample size. We found no association between hypertension comorbidity and presence of microbleeds in the dialysis cohort; four of four patients with microbleeds detected on MRI had history of hypertension vs. four of five patients with zero microbleeds at baseline and at follow-up.

## Discussion

In this paper, we report an increased prevalence of cerebral microhemorrhages in CKD mice and of microbleeds in CKD patients. CKD animals had evidence of BBB disruption, and cerebral microhemorrhages were increased over 2-fold compared with control mice. In the human study, ~ 50% of CKD patients (both in pre-dialysis and dialysis groups) had MRI-demonstrable microbleeds compared with 10% of controls. In our mouse studies, the highest microhemorrhage load was observed with LPS treatment in the adenine-CKD model, while development of microhemorrhages appeared to be independent of hypertension. The latter finding was consistent with our MRI studies of microbleeds in humans. Cell culture studies demonstrated significant impairment of the brain endothelial barrier upon exposure to uremic serum, with up to 65% drop in TEER in the 15% CKD serum group. Incubation with increasing concentrations of urea, the most abundant retained toxin in CKD [29], demonstrated disruption of the actin cytoskeleton and decreased tight junction proteins as potential mechanisms to explain the endothelial barrier dysfunction.

CKD has an impact on cerebrovascular disease risk that appears to go well beyond traditional risk factors such as hypertension and diabetes [10, 11]. Brain microbleeds are present in up to 50% of patients with advanced CKD [2–4] and correlate with cognitive dysfunction [4, 8, 19–23] and increased risk of hemorrhagic stroke [9]. Our group recently proposed CKD-specific pathways that could promote cerebral small vascular disease via disruption of blood flow autoregulation and BBB integrity, such as increased vascular calcification, systemic inflammation, and uremic toxins [1]. Our tissue culture studies demonstrate that exposure to urea in concentrations similar to those present in dialysis patients produced reduced expression of tight junction protein claudin-5 and disruption of the actin cytoskeleton. These pathways appear to explain, at least in part, the increased microhemorrhages due to increased BBB permeability observed in the CKD animals and patients. Of note, CKD has previously been associated with a marked depletion of tight junction proteins (75–80% decreased expression) in gut epithelial cells from experimental animals [30]. Subsequent in vitro studies revealed the central role of urea in the CKD-induced disruption of intestinal epithelial barrier structure and function [28]. Taken together, these observations suggest that urea toxicity is not limited to endothelial cells alone. Our data add to the growing body of evidence for direct adverse effects of uremic toxins on the vascular endothelium; other groups have reported that the gut-derived bacterial metabolites indoxyl sulfate and *p*-cresyl sulfate induce oxidative stress in cultured endothelial cells [31, 32].

LPS is an established acute inflammatory stimulus used in rodent models [33] that binds to toll-like receptor 4 [34] to induce BBB damage [35] and brain endothelial dysfunction [36, 37]. LPS has been used to amplify and study microbleeds pathophysiology in young adult and aged mice [25, 26, 38]. In our current animal studies, LPS treatment increased microhemorrhage formation to the same degree in CTL and CKD animals, by approximately 2-fold (Table 1; Fig. 2a). In CTL mice, average microhemorrhage count was increased from 2 to 3.6 per cm^2^ with LPS, whereas in CKD mice, the increase was from 4.5 to 10.3 per cm^2^. We conclude that the uremic brain is predisposed to more severe injury after an acute inflammatory event due to pre-existing injured BBB.

The actin cytoskeleton reversibly polymerizes between globular (G-actin) and filamentous (F-actin) configurations. F-actin is important for cell adhesion, division, and apoptosis; it is modulated by actin-binding proteins, which are in turn regulated by Rho GTPases [39, 40]. Actin reorganization, from its cortical distribution into stress fibers, is a key component of the endothelial response to inflammation [41]. Our cell culture studies implicated elevated urea in the pathogenesis of decreased F-actin expression, with fragmentation of cortical fibers and appearance of radial stress fibers (Fig. 4c). Further studies are needed to clarify the role of actin cytoskeletal disruption in microbleeds pathophysiology in vivo.

Although hypertension is a strong predictor of cerebral microbleeds in the general population [42], we found that hypertension did not modify presence of microbleeds in our CKD animal and human subjects (Tables 1 and 2). Our study is limited by small sample size; however, our results are consistent with prior cross-sectional reports in which CKD was a risk factor for brain microbleeds, independent of hypertension, age, and diabetes mellitus [10, 11]. The degree of hypertension in the 5/6 nephrectomy animals was mild; however, our findings are consistent with the study by Passos et al. in which induction of significant hypertension in wild-type mice via infusion of angiotensin II and L-N^G^-nitroarginine methyl ester (systolic 180 mmHg) did not significantly raise microhemorrhage counts [43]. While prevalence of brain microbleeds was similar in the two CKD patient groups (approximately 50%), analysis of consecutive brain MRIs demonstrated microbleed progression in three of four hemodialysis patients as compared to zero of four pre-dialysis patients (follow-up interval of ~ 1.5 years). Hemodialysis patients may be particularly predisposed to microbleed formation due to inter- and intradialytic BP fluctuations, regional gut ischemia leading to increased endotoxin translocation (which drives systemic inflammation), and use of heparin anticoagulation during dialysis therapy [1, 44, 45].

The major limitation of our cell culture work is the use of an immortalized cell line, with known limitation of brain-specific properties [46]. Further cell culture studies using, for example, primary brain endothelial cells are needed to confirm our findings. Our human study, a retrospective analysis of patients with serial brain MRIs, has the limitations of patient identification using hospital database diagnosis and procedure codes and of small sample size.

In summary, CKD increased brain microbleeds in both hypertensive and non-hypertensive mouse models. Uremic serum, and urea alone, disrupted the cultured brain endothelial cell monolayer, thus supporting a mechanistic role for uremic toxins affecting BBB permeability and promoting microhemorrhages. Human brain MRI studies confirmed increased prevalence of microbleeds in CKD patients compared with age-matched non-CKD controls, and hemodialysis patients in particular were noted to develop new microbleeds over a 1.5-year follow-up period. More studies are warranted to further characterize factors in the CKD milieu that promote brain microhemorrhages.

## References

[1] Lau WL, Huisa BN, Fisher M (2017). The cerebrovascular-chronic kidney disease connection: perspectives and mechanisms. Transl Stroke Res.

[2] Yokoyama S, Hirano H, Uomizu K, Kajiya Y, Tajitsu K, Kusumoto K (2005). High incidence of microbleeds in hemodialysis patients detected by T2*-weighted gradient-echo magnetic resonance imaging. Neurol Med Chir (Tokyo).

[3] Naganuma T, Takemoto Y, Yamasaki T, Shima H, Shoji T, Ishimura E, Nishizawa Y, Morino M, Okamura M, Nakatani T (2011). Factors associated with silent cerebral microbleeds in hemodialysis patients. Clin Nephrol.

[4] Chai C, Wang Z, Fan L, Zhang M, Chu Z, Zuo C, Liu L, Mark Haacke E, Guo W, Shen W, Xia S (2016). Increased number and distribution of cerebral microbleeds is a risk factor for cognitive dysfunction in hemodialysis patients: a longitudinal study. Medicine (Baltimore).

[5] Masson P, Kelly PJ, Craig JC, Lindley RI, Webster AC (2015). Risk of stroke in patients with ESRD. Clin J Am Soc Nephrol.

[6] Murray AM, Tupper DE, Knopman DS, Gilbertson DT, Pederson SL, Li S, Smith GE, Hochhalter AK, Collins AJ, Kane RL (2006). Cognitive impairment in hemodialysis patients is common. Neurology..

[7] Sarnak MJ, Tighiouart H, Scott TM, Lou KV, Sorensen EP, Giang LM, Drew DA, Shaffi K, Strom JA, Singh AK, Weiner DE (2013). Frequency of and risk factors for poor cognitive performance in hemodialysis patients. Neurology..

[8] Li L, Fisher M, Lau WL, Moradi H, Cheung A, Thai G, Handwerker J, Kalantar-Zadeh K (2015). Cerebral microbleeds and cognitive decline in a hemodialysis patient: case report and review of literature. Hemodial Int.

[9] Naganuma T, Takemoto Y, Shoji T, Ishimura E, Okamura M, Nakatani T (2015). Cerebral microbleeds predict intracerebral hemorrhage in hemodialysis patients. Stroke..

[10] Koren-Morag N, Goldbourt U, Tanne D (2006). Renal dysfunction and risk of ischemic stroke or TIA in patients with cardiovascular disease. Neurology..

[11] Ninomiya T, Perkovic V, Verdon C, Barzi F, Cass A, Gallagher M, Jardine M, Anderson C, Chalmers J, Craig JC, Huxley R (2009). Proteinuria and stroke: a meta-analysis of cohort studies. Am J Kidney Dis.

[12] Shoamanesh A, Kwok CS, Benavente O (2011). Cerebral microbleeds: histopathological correlation of neuroimaging. Cerebrovasc Dis.

[13] Fisher M, French S, Ji P, Kim RC (2010). Cerebral microbleeds in the elderly: a pathological analysis. Stroke..

[14] Bokura H, Saika R, Yamaguchi T, Nagai A, Oguro H, Kobayashi S, Yamaguchi S (2011). Microbleeds are associated with subsequent hemorrhagic and ischemic stroke in healthy elderly individuals. Stroke..

[15] Fazekas F, Kleinert R, Roob G, Kleinert G, Kapeller P, Schmidt R, Hartung HP (1999). Histopathologic analysis of foci of signal loss on gradient-echo T2*-weighted MR images in patients with spontaneous intracerebral hemorrhage: evidence of microangiopathy-related microbleeds. AJNR Am J Neuroradiol.

[16] Vernooij MW, van der Lugt A, Ikram MA, Wielopolski PA, Niessen WJ, Hofman A, Krestin GP, Breteler MMB (2008). Prevalence and risk factors of cerebral microbleeds: the Rotterdam Scan Study. Neurology..

[17] Fisher MJ (2013). Brain regulation of thrombosis and hemostasis: from theory to practice. Stroke..

[18] Kurella M, Chertow GM, Luan J, Yaffe K (2004). Cognitive impairment in chronic kidney disease. J Am Geriatr Soc.

[19] Poels MM, Ikram MA, van der Lugt A, Hofman A, Niessen WJ, Krestin GP (2012). Cerebral microbleeds are associated with worse cognitive function: the Rotterdam Scan Study. Neurology..

[20] van Es AC, van der Grond J, de Craen AJ, Westendorp RG, Bollen EL, Blauw GJ (2011). Cerebral microbleeds and cognitive functioning in the PROSPER study. Neurology..

[21] Akoudad S, Wolters FJ, Viswanathan A, de Bruijn RF, van der Lugt A, Hofman A, Koudstaal PJ, Ikram MA, Vernooij MW (2016). Association of cerebral microbleeds with cognitive decline and dementia. JAMA Neurol.

[22] Meier IB, Gu Y, Guzaman VA, Wiegman AF, Schupf N, Manly JJ, Luchsinger JA, Viswanathan A, Martinez-Ramirez S, Greenberg SM, Mayeux R, Brickman AM (2014). Lobar microbleeds are associated with a decline in executive functioning in older adults. Cerebrovasc Dis.

[23] Naganuma T, Takemoto Y (2015). New aspects of cerebrovascular diseases in dialysis patients. Contrib Nephrol.

[24] Yang HC, Zuo Y, Fogo AB (2010). Models of chronic kidney disease. Drug Discov Today Dis Models.

[25] Liu S, Grigoryan MM, Vasilevko V, Sumbria RK, Paganini-Hill A, Cribbs DH, Fisher MJ (2014). Comparative analysis of H&E and Prussian blue staining in a mouse model of cerebral microbleeds. J Histochem Cytochem.

[26] Sumbria RK, Grigoryan MM, Vasilevko V, Krasieva TB, Scadeng M, Dvornikova AK, Paganini-Hill A, Kim R, Cribbs DH, Fisher MJ (2016). A murine model of inflammation-induced cerebral microbleeds. J Neuroinflammation.

[27] Ekmark-Lewén S, Flygt J, Kiwanuka O, Meyerson BJ, Lewén A, Hillered L, Marklund N (2013). Traumatic axonal injury in the mouse is accompanied by a dynamic inflammatory response, astroglial reactivity and complex behavioral changes. J Neuroinflammation.

[28] Vaziri ND, Yuan J, Norris K (2013). Role of urea in intestinal barrier dysfunction and disruption of epithelial tight junction in chronic kidney disease. Am J Nephrol.

[29] Lau WL, Vaziri ND (2017). Urea, a true uremic toxin: the empire strikes back. Clin Sci (Lond).

[30] Vaziri ND, Yuan J, Rahimi A, Ni Z, Said H, Subramanian VS (2012). Disintegration of colonic epithelial tight junction in uremia: a likely cause of CKD-associated inflammation. Nephrol Dial Transplant.

[31] Stinghen AE, Chillon JM, Massy ZA, Boullier A (2014). Differential effects of indoxyl sulfate and inorganic phosphate in a murine cerebral endothelial cell line (bEnd.3). Toxins (Basel).

[32] Watanabe H, Miyamoto Y, Enoki Y, Ishima Y, Kadowaki D, Kotani S, Nakajima M, Tanaka M, Matsushita K, Mori Y, Kakuta T, Fukagawa M, Otagiri M, Maruyama T (2015). P-Cresyl sulfate, a uremic toxin, causes vascular endothelial and smooth muscle cell damages by inducing oxidative stress. Pharmacol Res Perspect.

[33] Juskewitch JE, Knudsen BE, Platt JL, Nath KA, Knutson KL, Brunn GJ, Grande JP (2012). LPS-induced murine systemic inflammation is driven by parenchymal cell activation and exclusively predicted by early MCP-1 plasma levels. Am J Pathol.

[34] Nagyoszi P, Wilhelm I, Farkas AE, Fazakas C, Dung NT, Haskó J (2010). Expression and regulation of toll-like receptors in cerebral endothelial cells. Neurochem Int.

[35] Banks WA, Gray AM, Erickson MA, Salameh TS, Damodarasamy M, Sheibani N, Meabon JS, Wing EE, Morofuji Y, Cook DG, Reed MJ (2015). Lipopolysaccharide-induced blood-brain barrier disruption: roles of cyclooxygenase, oxidative stress, neuroinflammation, and elements of the neurovascular unit. J Neuroinflammation.

[36] Munshi N, Fernandis AZ, Cherla RP, Park IW, Ganju RK (2002). Lipopolysaccharide-induced apoptosis of endothelial cells and its inhibition by vascular endothelial growth factor. J Immunol.

[37] Karahashi H, Michelsen KS, Arditi M (2009). Lipopolysaccharide-induced apoptosis in transformed bovine brain endothelial cells and human dermal microvessel endothelial cells: the role of JNK. J Immunol.

[38] Sumbria RK, Grigoryan MM, Vasilevko V, Paganini-Hill A, Kilday K, Kim R, Cribbs DH, Fisher MJ (2018). Aging exacerbates development of cerebral microbleeds in a mouse model. J Neuroinflammation.

[39] Lai J.-M. (2003). Caspase activation during phorbol ester-induced apoptosis requires ROCK-dependent myosin-mediated contraction. Journal of Cell Science.

[40] Stricker J, Falzone T, Gardel M (2010). Mechanics of the F-actin cytoskeleton. J Biomech.

[41] Prasain N, Stevens T (2009). The actin cytoskeleton in endothelial cell phenotypes. Microvasc Res.

[42] Romero JR, Preis SR, Beiser A, DeCarli C, Viswanathan A, Martinez-Ramirez S, Kase CS, Wolf PA, Seshadri S (2014). Risk factors, stroke prevention treatments, and prevalence of cerebral microbleeds in the Framingham Heart Study. Stroke..

[43] Passos GF, Kilday K, Gillen DL, Cribbs DH, Vasilevko V (2016). Experimental hypertension increases spontaneous intracerebral hemorrhages in a mouse model of cerebral amyloidosis. J Cereb Blood Flow Metab.

[44] McIntyre CW, Harrison LE, Eldehni MT, Jefferies HJ, Szeto CC, John SG (2011). Circulating endotoxemia: a novel factor in systemic inflammation and cardiovascular disease in chronic kidney disease. Clin J Am Soc Nephrol.

[45] Shen JI, Winkelmayer WC (2012). Use and safety of unfractionated heparin for anticoagulation during maintenance hemodialysis. Am J Kidney Dis.

[46] He Y, Yao Y, Tsirka SE, Cao Y (2014). Cell-culture models of the blood-brain barrier. Stroke..

